# The Effect of Synovial Fluid Composition, Speed and Load on Frictional Behaviour of Articular Cartilage

**DOI:** 10.3390/ma13061334

**Published:** 2020-03-15

**Authors:** Denis Furmann, David Nečas, David Rebenda, Pavel Čípek, Martin Vrbka, Ivan Křupka, Martin Hartl

**Affiliations:** Faculty of Mechanical Engineering, Brno University of Technology, Brno 616 69, Czech Republic; David.Rebenda@vut.cz (D.R.); Pavel.Cipek@vut.cz (P.Č.); Martin.Vrbka@vut.cz (M.V.); krupka@fme.vutbr.cz (I.K.); Martin.Hartl@vut.cz (M.H.)

**Keywords:** biotribology, articular cartilage, coefficient of friction, tribological properties, synovial fluid

## Abstract

Articular cartilage ensures smooth motion of natural synovial joints operating at very low friction. However, the number of patients suffering from joint diseases, usually associated with cartilage degradation, continuously increases. Therefore, an understanding of cartilage tribological behaviour is of great interest in order to minimize its degradation, preserving the reliable function of the joints. The aim of the present study is to provide a comprehensive comparison of frictional behaviour of articular cartilage, focusing on the effect of synovial fluid composition (i), speed (ii), and load (iii). The experiments were realized using a pin-on-plate tribometer with reciprocating motion. The articular cartilage pin was loaded against smooth glass plate while the tests consisted of loading and unloading phases in order to enable cartilage rehydration. Various model fluids containing albumin, γ-globulin, hyaluronic acid, and phospholipids were prepared in two different concentrations simulating physiologic and osteoarthritic synovial fluid. Two different speeds, 5 mm/s and 10 mm/s were applied, and the tests were carried out under 5 N and 10 N. It was found that protein-based solutions exhibit almost no difference in friction coefficient, independently of the concentration of the constituents. However, the behaviour is considerably changed when adding hyaluronic acid and phospholipids. Especially when interacting with γ-globulin, friction coefficient decreased substantially. In general, an important role of the interaction of fluid constituents was observed. On the other hand, a limited effect of speed was detected for most of the model fluids. Finally, it was shown that elevated load leads to lower friction, which corresponds well with previous observations. Further study should concentrate on specific explored phenomena focusing on the detailed statistical evaluation.

## 1. Introduction

Joint osteoarthritis (OA) represents one of the most common diseases all over the world. It is estimated that more 50% of the adult population in the US will be diagnosed with joint OA by 2040 [1]. Arthritis is a degenerative disease of articular cartilage, usually occurring in large synovial joints such as hips and knees [2]. Nevertheless, it is reported that the occurrence of arthritis increases also in the case of small joints such as elbows, wrists, or fingers [3,4]. OA mainly affects older people [5] and its presence gradually grows with age. It is characterized by a strong joint pain which often leads to movement disabilities. Because of the avascular and aneural nature of the articular cartilage, there are limited possibilities of curing OA [6]. One of the options is to apply hyaluronic acid (HA)-based viscosupplements. However, the efficiency is not guaranteed and the treatment effect often lasts for a limited time [7]. Therefore, the only chance for the patients suffering from OA is often represented by the joint replacement, the number of which gradually increases with rapid growth reported within the last decade [8]. To prevent further substantial increases in the number of joint replacements, preventing the degradation of articular cartilage seems to be a challenging task for upcoming decades. In that case, knowledge of the tribological properties of synovial fluid constituents and their interactions could help to develop new therapeutic procedures for the treatment of OA.

The main function of the articular cartilage is to ensure the transfer of load during movement while keeping a very low coefficient of friction [9]. The joints operate in several lubrication regimes, dependent on the loading conditions. The tribological performance of articular cartilage is mostly associated with its bi-phasic character [10]. Solid-phase consists mostly of the collagen fibres and proteoglycans, while the liquid phase is represented by the water and dissolved salt ions [6]. The biphasic character of lubrication was described in [11]. The authors showed that the liquid bound to the cartilage tissue is able to transfer part of the load. In the case of permanent load, the liquid is pushed out of the contact zone to the unloaded regions. A minor portion of the load is being supported by the liquid, and the gradual increase in friction is measured. The lubrication mode steadily changes from the so-called biphasic to boundary.

The dependence of friction on time was demonstrated by Forster and Fisher [12,13]. It was shown that the value of the friction factor grows until the whole load is transmitted only by a solid phase of the cartilage. Thus, the ability to rehydrate pushed liquid back to the previously loaded tissue significantly affects lubrication performance. However, rehydration itself is influenced by sliding speed [14] or whether the contact point is moved to another location [15]. Since the lubrication regime depends not only on the composition of the lubricant but also on the structure of the cartilage, several studies analysed the effect of the degradation of the cartilage tissue. Bell et al. [16] analysed the effect of the collagen network disruption. It was shown that the damaged cartilage samples reached higher values of the friction coefficient.

The region between contact pairs is filled with the synovial fluid (SF), which also acts as a boundary lubricant [15]. Recent studies analysed the adsorption properties of synovia constituents. It was shown that proteins significantly affect the adsorption of the HA [17,18,19]. Synergic effect of γ-Globulin and HA was further observed. γ-globulin adsorbs on the cartilage surface while HA adsorbs on the protein layer, forming stable lubricating film exhibiting lower friction. However, such an effect was not measured when using albumin and HA. An amount of studies was aimed at determining the constituents responsible for lubricating properties. Hills and Buttler [20] studied the effect of enzymatic degradation of various constituents. No significant change in friction was observed with the dissolving HA. The strongest impact was observed with the degradation of phospholipids (PHs). However, a slight improvement in lubrication properties was observed in terms of the degradation of the proteins. On the contrary, Bell et al. [21] examined the effect of the concentration of the ions in the salt solution and HA, with significant improvement in tribological properties being observed when adding HA to the solution. Forsey et al. [22] examined the impact of HA and PHs. It was shown that the efficiency of HA was not dependent on the concentration. However, the concentration had a major impact on the tribological properties of PHs. In the following study [23], the influence of PHs and proteins was observed, showing a slight increase in the friction coefficient for lipid and protein-impaired cartilage compared to the reference cartilage sample. The differences were apparent especially at shorter loading times. The effect of lubricin was analysed in the upcoming study [24], in which the synovial fluid was collected from the individuals with decreased lubricin presence. Synovial fluid with less lubricin had much worse tribological properties compared to the synovial fluid of a healthy individuals. Moreover, Ludwig et al. [25] and Schmidt et al. [26] showed that the combination of lubricin, HA and PHs results in a synergic improvement of the tribological properties.

It was shown above that extensive research has been carried out regarding articular cartilage behaviour. However, with respect to limited abilities of cartilage treatment, various authors aimed at the possibility of the cartilage replacement in recent years. In that case, polyvinyl alcohol hydrogel (PVA) seems to be a suitable anticipating material [27]. Recently, several studies have focused on the analysis of PVA hydrogel tribological properties. Hydrogel has similar properties to the natural cartilage [17]. The impact of the individual components of synovial fluid on friction was investigated. It was found that the constituents in contact with the glass plate had the same effect as in natural cartilage. The effect of the adsorbed protein layer was studied in [18] concluding that the adsorbed proteins have a significant effect on friction. In particular, γ-globulin led to reduction of friction value. However, in the following study [28], where the separate impact of the constituents was evaluated, the effect of γ-globulin was shown to be the opposite. In the next paper [29], the effect of a PHs in combination with proteins was investigated. It was shown, that the concentration of PHs has a major impact on the tribological properties. In the studies led by Klein [30,31,32], it was shown that the presence of proteoglycans has a positive effect on the frictional properties, and that friction depends on the type of the PH used when combined with HA.

Based on the above references, it can be concluded that articular cartilage has been extensively investigated over the last few decades. Various studies focusing on cartilage behaviour may be found; however, there is a lack of complex frictional assessment so far. Therefore, the aim of the present study is to provide a comprehensive analysis of the frictional response of articular cartilage considering the effect of synovial fluid composition, speed, and load. The loading and kinematic conditions are designed based on literature in order to mimic common daily activities (slow, normal walking, stair climbing—elevated load). In addition, since it is assumed that synovial fluid composition plays a major role in joint lubrication, the main attention is paid to the concentration and mutual interactions of individual synovial fluid constituents. The present study should point to the fundamentals of cartilage friction. Future study should concentrate on specific observed phenomena, focusing on the improved statistical evaluation.

## 2. Material and Methods

**Figure 1:**
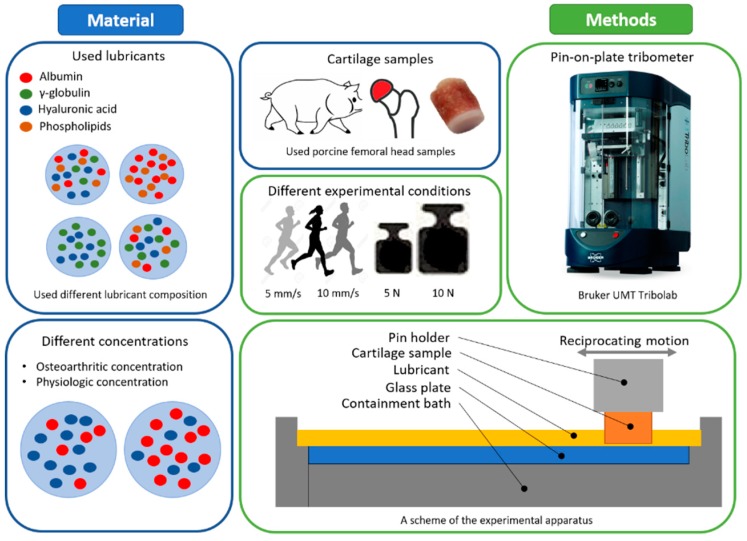
A research plan of the study.

The experiments were carried out in pin-on-plate configuration using commercial tribometer Bruker UMT TriboLab (Bruker, Billerica, MA, USA). The contact of porcine articular cartilage pin and smooth glass plate was investigated. The cartilage specimens were prepared from a porcine femoral head. The bones were collected from a local butchery shortly after slaughtering of six to eight months old mature pigs having a mass around 100–120 kg. Pins of a diameter of 5.6 mm were cored out using a hollow drill from the femoral heads. The subchondral bone was retained along with the cartilage tissue for proper fixation during the friction tests. After preparation, cartilage pins were stored in the phosphate buffered saline (PBS) solution for one hour in order to ensure sufficient tissue hydration, and were then frozen at −22 °C until further use. Optical glass plates were used, since the nature of glass mimics the superficial layer of articular cartilage under wet conditions, as discussed below. The tests were conducted at two different sliding speeds of 5 mm/s and 10 mm/s, representing slow and normal walking conditions. To explore the role of load, two different levels of normal force were considered; 5 N and 10 N, in particular. Following the elastic properties of the articular cartilage [33], the glass (technical datasheet), and the curvature of the cartilage pin, the contact pressure should correspond to approximately 0.3 to 0.5 MPa. Similar load was applied in previous references dealing with cartilage investigation [34,35]. Due to the soft nature of the articular cartilage, the size of the contact area corresponds to the whole pin contact surface. In all the tests, the stroke length of 20 mm was used. The research plan is schematically shown in Figure 1. Initially, the tests under the given conditions with six randomly chosen model fluids (no. 2, 4, 6, 10, 12, 13, see Table 1) were performed two times in order to check the repeatability of the experiments. As satisfactory compliance of the data was observed together with confirmation of some expectations based on the literature, the rest tests were performed only once following the below described procedure. However, in the case of any doubt, the experiments were repeated again in order to confirm the validity of the conclusions.

**Figure 2:**
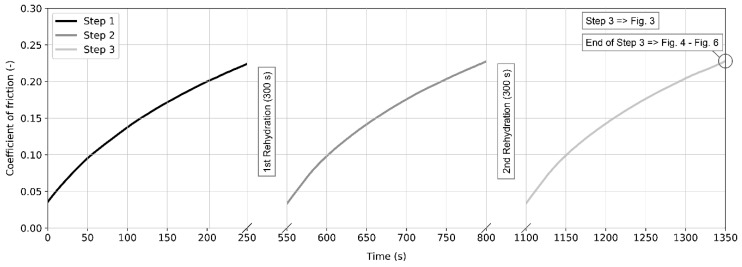
A schematic illustration of the test procedure.

**Figure 3:**
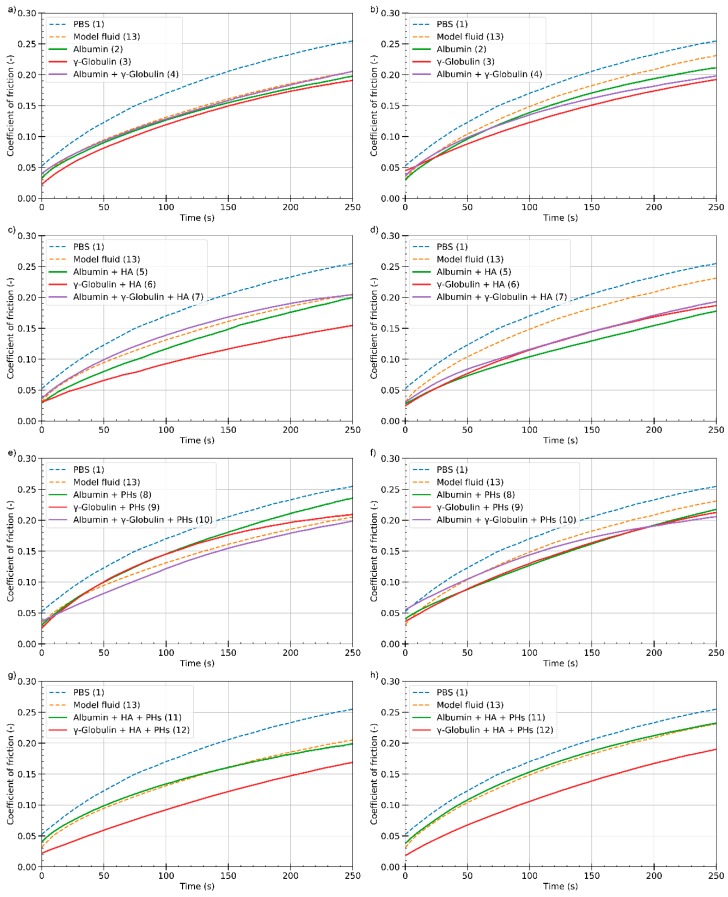
Dependence of the friction coefficient on time for various protein- (**a**,**b**), HA- (**c**,**d**), (PHs)- (**e**,**f**), and HA + PHs- (**g**,**h**) based lubricants; PC (**left**) and OC (**right**). HA: hyaluronic acid; PH: phospholipids; PC: physiologic concentration; OC: osteoarthritic concentration.

**Figure 4:**
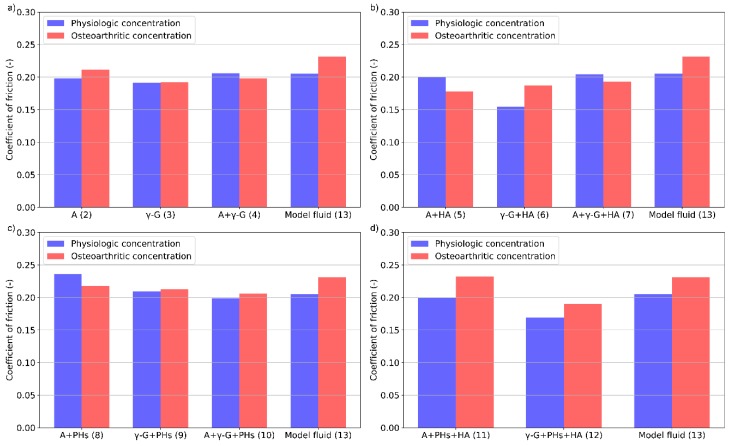
The effect of concentration on the friction coefficient for various protein- (**a**), protein + HA- (**b**), PHs- (**c**), and HA + PHs- (**d**) based lubricants. (A—Albumin, γ-G—γ-Globulin).

**Figure 5:**
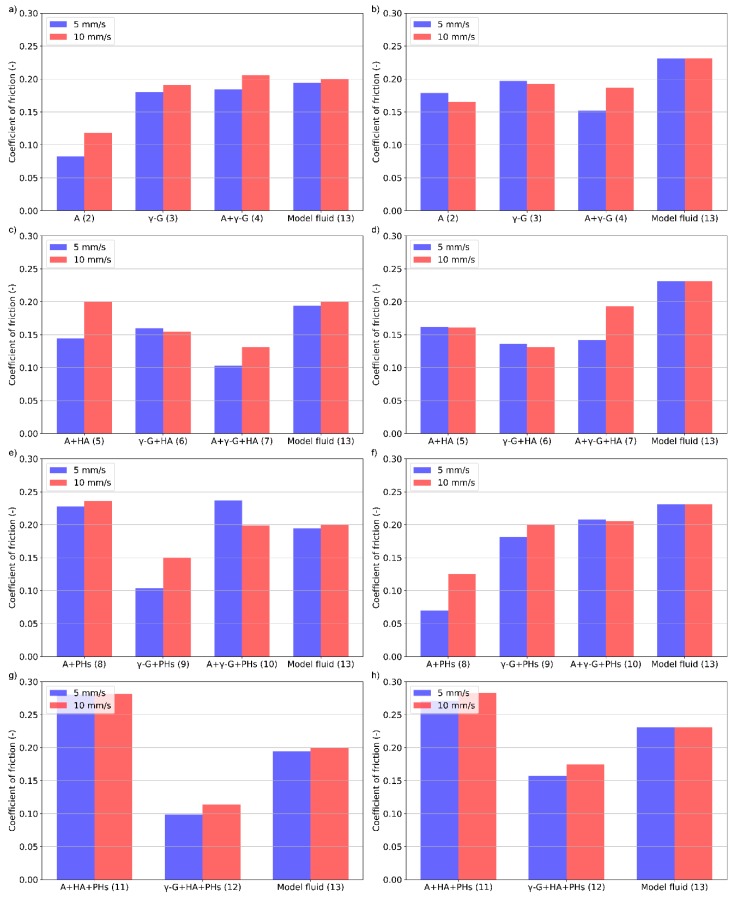
The effect of speed on the friction coefficient for various protein- (**a**,**b**), HA- (**c**,**d**), PHs- (**e**,**f**), and HA + PHs- (**g**,**h**) based lubricants; PC (**left**) and OC (**right**).

**Figure 6:**
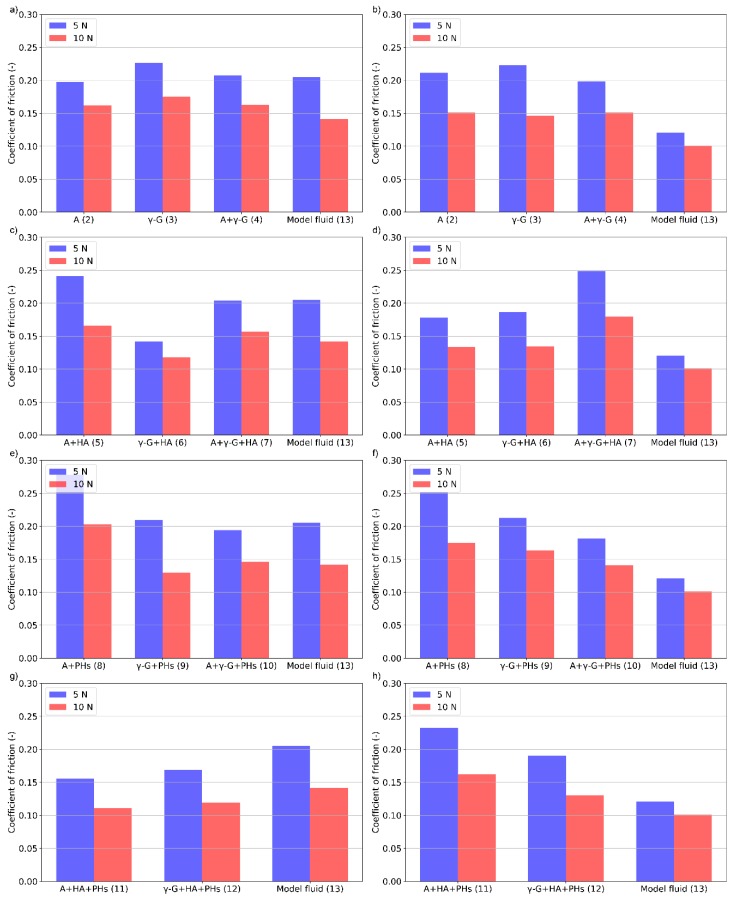
The effect of load on the friction coefficient for various protein- (**a**,**b**), HA- (**c**,**d**), PHs- (**e**,**f**), and HA + PHs- (**g**,**h**) based lubricants; PC (**left**) and OC (**right**).

Before the test, the glass plate was cleaned using a sodium dodecyl sulphate solution and isopropyl alcohol and then dried by pressed air. The lubricants and the cartilage specimens were thawed at room temperature for 30 min and were stored in a fluid bath to avoid dehydration. Each experiment consisted of five steps—three friction (loading) steps and two unloading steps in order to enable cartilage rehydration [18,28]. Focusing on daily activities such as walking, the articular cartilage is repeatedly loaded and unloaded within very short time intervals. This is difficult to simulate using a laboratory tester. Therefore, each loading phase lasting 250 s was followed by 300 s unloading. Therefore, the overall duration of the experiment was 22.5 min (12.5 min of friction steps and 10 min of rehydration). The schematic illustration of the test procedure is shown in Figure 2. Focusing on Figure 3, the displayed data correspond to the third (last) friction step. The values of friction presented in Figure 4, Figure 5 and Figure 6 correspond to the friction at the end of the third test.

In the present study, various model solutions were tested. PBS was used as a base fluid, while albumin, γ-globulin, HA, and PHs were added. The constituents were initially separately solved with PBS overnight at 4 °C using laboratory rocker-shaker (MR-12, Biosan, Riga, Latvia). Subsequently, the individual solutions were mixed together in order albumin, γ-globulin, HA, PHs. Focusing on the specific constituents, the following products were used. Bovine serum albumin (powder, ≥96%; A2153, Sigma-Aldrich, St. Louis, MO, USA), γ-globulin from bovine blood (powder, ≥99%; G5009, Sigma-Aldrich, St. Louis, MO, USA), HA = Sodium Hyaluronate HySilk (powder, quality class—cosmetic; molecular weight = 820–1020 kDa, Contipro, Dolní Dobrouč, Czech Republic), and PHs = L-α-Phosphatidylcholine (powder, Type XVI-E, lyophilized powder; ≥99%; vesicles form; P3556, Sigma-Aldrich, St. Louis, MO, USA). After preparation, the lubricants were frozen at −22 °C until further use. The fluids of two different concentrations were prepared, representing both healthy (physiologic) and osteoarthritic synovial fluid concentrations [36]. The combinations of the chosen lubricant constituents can be seen in Table 1, and the used concentrations in Table 2.

## 3. Results

### 3.1. The Effect of Fluid Composition

The results of friction coefficient during the third step of the test for various lubricants are summarized in Figure 3. The results for lubricants of physiological concentration (PC) of the constituents are shown in the left, while osteoarthritic concentration (OC) fluids are shown in the right figures. As can be seen in Figure 3, the values of the friction coefficient for pure PBS reach the highest values for the whole duration of the experiment. Thus, the constituents always led to lower friction. It is also evident from the figure that the model synovial fluid does not always reach the lowest friction.

For the lubricants at PC, it is seen that the values of the friction coefficient are very similar to those of a model synovial fluid during the test for protein-based lubricants (Figure 3a). The differences are small, and it is not possible to clearly assess which lubricant leads to better performance. In the case of a concentration typical for patients suffering from osteoarthritis, the model fluid has inferior tribological properties than for the PC. However, for the lubricants containing only the proteins, only a slight change of the friction coefficient is observed. All the observed lubricants achieved lower values of friction than pure PBS.

Addition of HA does not considerably change the results compared to the protein solution lubricants (Figure 3c,d). In the case of a PC (Figure 3c), the lowest values of friction were achieved with the γ-globulin and HA. However, in the case of albumin and both proteins mixtures with HA, no visible change occurred. Similar to the protein solution lubricants without the addition of HA, these lubricants have achieved comparable values as the model fluid. At OC (Figure 3d) a similar result is observed. Likewise, as in the case of the protein solution lubricants, these achieved lower values of friction than a model fluid. When comparing the values of the friction coefficient with lubricants containing only proteins, a slight decrease in friction is seen when the HA is added indicating positive lubricating effect of HA.

A further set of the experiments was conducted with the lubricants based on PHs (Figure 3e,f). At the PC (Figure 3e), it can be seen that the addition of PHs does not have a synergic effect in most cases. In the case of γ-globulin and albumin containing lubricants, the gradual growth of the friction is almost the same for the first 120 s. However, in the case of albumin, the growth continues during the test, and the end value is a bit higher. In the case of a combination of proteins, the value is similar to the model fluid. Results of OC fluids are shown in Figure 3f. Focusing on the combination of albumin and γ-globulin, although the initial value is higher than for the model fluid, friction growth is less steep than that of the model fluid. For sole protein lubricants, the value of friction is lower than that of the model fluid during the test. Moreover, the growth curves are steeper than that of the lubricant containing both the proteins. There is a cross point at around 200 s when the friction of simple protein solutions exceeds the friction of mixed solution. Nevertheless, the observed friction is always lower than that of the model fluid.

When HA and PHs are present in the lubricant, similar results are seen at both concentrations (Figure 3g,h). The growth curves are similar for model fluid and the albumin lubricants. The values start at the same value at each concentration. However, at OC, the growth is steeper and the friction reaches higher values. The lowest friction is observed for the γ-globulin lubricant during the test. Likewise, the growth is steeper at the OC.

### 3.2. The Effect of Concentration

It is evident from Figure 4b that HA with γ-globulin has a synergic effect. At PC, it achieves the lowest friction for all the investigated lubricants. However, when the concentration of HA was lower (OC), there was an obvious increase of friction similar to that of the model fluid. Nevertheless, in the case of a combination of proteins or simple albumin with HA, only a limited change of friction is observed.

In the case of addition of PHs to the solutions (Figure 4c), a very slight decrease in friction can be seen for the lubricants containing albumin. During the test, this lubricant exhibited the highest values of the friction coefficient. When the concentration was altered, the concentration of both the components (albumin, PHs) decreased; therefore, it is evident that their combination affects the frictional behaviour. On the contrary, no change of friction is observed when γ-globulin or combination of albumin and γ-globulin was used. For HA and PHs-based lubricants (Figure 4d), a major increase of friction is observed for all the analysed lubricants. Values of friction are similar at each concentration for model fluid and albumin lubricant. However, when only γ-globulin was used, a lower value of friction was observed. Furthermore, the friction was the lowest from all the observed lubricants during the test.

### 3.3. The Effect of Speed

For a complex model fluid, a negligible impact of speed is observed independently of concentration, as is shown in Figure 5. On the other hand, there is a visible effect of speed for albumin-based lubricant. By lowering the speed to 5 mm/s, a decrease in friction is observed at PC, but there is a slight increase at OC for single protein lubricants. For pure γ-globulin, the effect of speed seems to be not as important. At PC, a little improvement of the friction is observed, but at OC a small increase of the friction is evident. However, compared to albumin, these changes are much lower. For combination of the proteins, the change in the values at both the studied concentrations is similar. In both cases, a slight reduction in the friction coefficient occurs when the speed decreases. The results for protein-based lubricants under two different speeds are shown in Figure 5a,b.

In the case of addition of HA to albumin, the impact of speed is very similar to the pure albumin (Figure 5c,d). At the PC, an apparent decrease of friction is observed when the speed is lowered, but no effect is observed at the OC. For γ-globulin, a very small increase in the coefficient of friction is observed at both concentrations. However, compared to the albumin, this increase is negligible. Also, albumin with γ-globulin has a similar effect at both observed concentrations. With a reduction in speed, an evident reduction of the friction coefficient occurs.

Addition of PHs led to opposite speed effect for albumin solution at OC (Figure 5f). As is displayed in the figure, with the decrease of speed, a substantial reduction of the coefficient of friction occurs. However, at PC, only a minimum effect is observed (Figure 5e). The effect of PHs on pure γ-globulin is the opposite. For the combination of albumin and γ-globulin, a slight increase of friction is observed at PC; however, no effect is observed at the OC. For the combination of HA and PHs-based lubricants (Figure 5g,h), a decrease in friction is observed with the reduction of the speed for the γ-globulin containing lubricant. However, there is no considerable change in the friction coefficient for the model fluid and the albumin-containing solution.

### 3.4. The Effect of Load

Based on Figure 6, it can be concluded that the impact of load on friction is the same for all the lubricants and concentrations. Only the difference in terms of percentage change can be identified. Independently of the test lubricant, lower friction coefficients were observed for higher levels of load. Figure 6a,b shows the load dependence for lubricants containing only the proteins. The most apparent friction reduction occurs at both the concentrations when simple γ-globulin solution is applied. In particular, at OC, the decrease is more pronounced. Likewise, the decrease is more pronounced at OC when the lubricant contains only albumin. A slight change is observed for lubricants containing the mixture of albumin and γ-globulin. The decrease in friction for the complex PC is considerable; nevertheless, very little effect is observed at OC where the difference in friction values is the lowest.

After adding HA to the protein solutions (Figure 6c,d), the greatest decrease of friction is observed for albumin containing lubricant at PC. On the contrary, for the lubricant with γ-globulin, only a negligible effect is achieved. For a combination of proteins, the decrease is similar to that of a model fluid. At OC, the most apparent decrease is observed for a combination of proteins. For the lubricants containing only one of the proteins, the observed effect is similar but much lower than that of the combination of the proteins.

Figure 6e,f show the effect of load considering the lubricants with the addition of PHs. At PC, a major reduction of friction is observed for single protein lubricants. For a combination of proteins, the decrease is slightly lower than that of the model fluid. Contrary, at OC the most considerable decrease of friction is observed for the lubricant containing albumin. The following are a γ-globulin and the combination of proteins. The decrease of these is similar; however, it is more pronounced than for the OC. The impact of the load with the addition of both HA and PHs is shown in Figure 6g,h. The most considerable decrease considering PC lubricants is observed for the complex model fluid. However, at the OC, the decrease of friction is the lowest for the model fluid. The decrease of friction for the single protein lubricants is similar at PC.

## 4. Discussion

The results show that the friction coefficient strongly depends on both the used composition and the selected lubricant concentration. In the case of lubricants containing only proteins, the limited effect of the concentration or the composition is observed. Each lubricant reached approximately the value of the friction coefficient at around 0.2. The effect of HA or PHs has been analysed in several studies. Seror and co-workers, as shown in the works [30,31], used avidin, which is a protein extracted from egg whites. Murakami et al. [17,18,28,29] predominantly examined the characteristics of the hydrogel applying different concentrations of the constituents. However, compared to the present results, their chosen concentrations were one order of magnitude lower than the concentrations used at the present study.

The quantity of each constituent was chosen to represent the actual concentrations occurring in the human body. Study [28] used the same set of lubricants; however, the concentrations employed were lower by approximately one order of magnitude. With the use of such low concentrations, a substantial effect of the proteins on lubrication properties was observed. However, when using a higher amount of protein, as was used in the present study, no difference in the lubrication properties was demonstrated. Proteins are adsorbed onto the surface of the articular cartilage and create a boundary lubricating film. According to Figure 3, the lubricating capacity seems to be not dependent on the concentration or the used protein. It rather looks like each protein is sufficient in order to create a lubricating film. Moreover, the same effect was observed when both the proteins were used. Proteins that do not contribute to the formation of the lubrication film probably do not have any substantial effect on friction. In reference [37], the authors analysed the adsorption properties of the proteins on the cartilage surface. It was shown that γ-globulin forms a more stable lubricating film than albumin, and the values of friction should therefore be different from each other. However, as stated before, no such effect was observed. It is assumed that this influence is caused primarily by the different concentrations of the proteins.

In Table 2, it can be seen that the percentage change of concentration (considering PC and OC) is not the same for all the constituents. Therefore, in order to study the effect of HA or PHs, concentration changes were mostly taken to be related to the concentration of the protein at each chosen concentration set. For lubricants with γ-globulin, Figure 4 shows a rapid increase of friction that is dependent on the concentration of HA. The synergic effect of these components is thus evident. However, when focusing on albumin and a combination of proteins, higher friction is observed at a higher relative concentration of HA. In addition, HA seems to have a negative effect on the adsorption of albumin on the articular cartilage surface. This finding is in compliance with previous observations [38].

The concentration of PHs and γ-globulin is higher in the case of OC. However, the relative concentration of these constituents is very similar. In Figure 4, no change of the friction coefficient for lubricants containing γ-globulin as well as a combination of proteins was observed. The friction value is similar to that when only protein containing lubricants are used. On the contrary, it is apparent that the combination of albumin and PHs at PC and OC does not form a sufficient boundary lubricant. The friction level at PC is only slightly lower than that of PBS. Although the friction at OC is lower, the value is still higher than lubricant containing only albumin. In the study [29], it was shown that addition of PHs is not always necessarily associated with the reduction of friction. Furthermore, in [22] it was demonstrated that friction is significantly affected by the concentration of the PHs and proteins.

In the case of combination of HA and PHs, it is evident from Figure 4 that each lubricant exhibited slightly elevated values of the friction coefficient at OC. In general, the lowest friction was achieved with the lubricant containing γ-globulin. However, the lubricants where albumin was presented achieved a similar friction as the model fluid at both concentrations. In the previous reference [29], the lowest friction was detected for the combination of all the components of the model fluid. It is therefore likely that, as is the case with the addition of PHs, friction is strongly dependent on the concentration of the proteins in the lubricant.

Focusing on the effect of speed, a very interesting similarity between protein-based and HA-based lubricants is observed. The percentage change is not as considerable for the lubricant with γ-globulin, as is also the case with the model fluid. However, it is evident from Figure 5 that for lubricants containing albumin, the magnitude of the effect of speed depends on the concentration. The impact of speed has been studied in the literature previously [14]. The authors found that the coefficient of friction depends on the concentration of salt ions. A similar dependence was observed with the albumin containing lubricant, even if HA is added. For lower concentrations, which is a PC for albumin, the increase of friction is observed with increased speed. On the contrary, when albumin concentration is increased to OC, which represents growth by around 20%, a slight lowering of friction can be seen in Figure 5. It is likely that the adsorbed protein layer and HA have no effect on speed, and thus the whole effect is caused by the present charge of albumin molecules. However, when HA and PHs are added, the limited effect of speed is observed. PHs are also charged molecules; however, the lubrication mechanism is different. When only PHs are added to the albumin solution, a strong effect of speed is observed only at OC. At the PC, a small increase of friction is observed; however, it is much less pronounced as in the case of OC. The effect thus apparently depends on which proteins and which concentration is considered, similar as it is with the effects of composition and concentration. The findings are in accordance with the previous observations [29], where strong influence of concentration was observed as well.

It is shown that with increasing load, the friction coefficient decreases for all the applied lubricants. The impact of load was previously explained by Walker et al. [39]. Under a constant load, the fluid is slowly pushed into a region where the cartilage is not loaded. However, the porous structure of the articular cartilage causes high resistance which is accompanied by the increase of pressure inside of the cartilage tissue. A higher load causes higher pressure, and thus the liquid phase can transfer a larger portion of the load. That explains the decrease of the friction coefficient. The decrease is thus caused by the structure of the cartilage and not by the properties of the lubricant. It can be assumed that the level of friction reduction may be related to the thickness and permeability of the lubricating film. Therefore, the creation of boundary lubricating film affects only the rate of the friction drop.

The authors recognize several drawbacks of the performed study. The most important point is related to measurement repeatability. Since the articular cartilage is a biological tissue, the repeatability is usually not as good as in the case of technical materials. The main motivation for the performed study was to provide a very complex comparison of cartilage frictional behaviour focusing on the effect of speed, load, and synovial fluid composition. It is generally recommended to provide seven repetitions at minimum for biological samples. When summarizing all the studied conditions and fluids, this would lead to nearly 600 separate experiments, each lasting nearly 23 min. Clearly, this could not be done due to (i) amount of cartilage samples, (ii) costs of model fluids, and (iii) required time for testing. However, it should be pointed out that the authors followed very strict experimental procedure. The cartilage specimens were extracted from the same parts of the porcine femoral bones collected shortly after slaughtering from a local butcher. In the case of any suspicion about the condition of the bone, or extraction of the cartilage pin, these samples were immediately excluded. At the beginning of the study, six random lubricants were chosen, and the experiments were repeated two times in order to check the repeatability. As very satisfactory compliance was observed, further measurements were realized only once. Nevertheless, the results of the tests were analysed independently by various members of the research team in order to prevent unclear conclusions. If necessary, the experiments under some conditions were repeated more times. Therefore, even if the authors could not provide extensive statistical analysis in the present study, it is assumed that the findings are relevant showing clear findings and dependencies. This statement is supported by a comprehensive data set with clear effects across a wide range of lubricants and experimental conditions. In addition, excellent repeatability could be obtained for three consequence friction tests performed with each specimen, as illustrated in Figure 2. According to the author’s best knowledge, such a complex study has not been introduced before. Our further motivation is to combine the information about frictional behaviour with direct in situ observation of the contact using fluorescent microscopy method [40]. This technique makes it possible to qualitatively assess the film thickness together with the determination of the role of individual synovial fluid constituents [41]. Moreover, the main motivation for the present study was to provide a comprehensive assessment of the cartilage frictional behaviour. Specific observed phenomena are going to be investigated in the upcoming study. This should focus on specific conditions and fluids, concentrating on fully relevant statistical description.

Focusing on the previous research, it was found that human synovial fluid exhibits significant differences in viscosity [36]. However, it should be emphasized that all the fluids employed in the present study were prepared artificially from the same constituents of the same series provided by the same producers. Although there is an apparent difference in content of individual parts regarding physiologic and osteoarthritic fluid, the viscosity difference was found to be negligible varying from 2.6 to 2.8 mPa·s for PC and OC, respectively. Therefore, it is assumed that the difference in friction corresponds to interaction of the constituents rather than to the effect of viscosity.

A further limitation of the study is related to the contact mechanics. The present paper uses cartilage-on-glass contact. It is particularly complicated to investigate real cartilage-on-cartilage contact in the laboratory. The point is that it is nearly impossible to get a flat cartilage sample of sufficient size in order to enable mutual motion in the range of a couple of millimetres at least. Therefore, one of the rubbing surfaces is often substituted by technical material [12,13,23,28]. Glass as a counter face was used by Murakami et al. [28], among others. It has been discussed in the literature that selection of glass may benefit from its properties. Glass is generally very smooth, hard, nonporous, and impermeable, compared to articular cartilage; however, it exhibits a hydrophilic negatively charged nature, corresponding to proteoglycan on superficial layer of articular cartilage under wet conditions [42]. Thus, glass is considered to be a suitable counterpart material. Further motivation for using glass is related to the possibility of visualization of lubricant film formation [43] using optical fluorescent microscopy [40] in future. The results will be confronted in the upcoming study. Moreover, it should be emphasized that glass apparently has a very limited effect on frictional behaviour. This could be clearly seen during the experiments. After rehydration, friction dropped to the same level as was at the beginning of the test; indicating that there were no molecules adsorbed on the glass surface which was flooded all the time.

When summarizing the drawbacks of the paper, simplified loading and kinematic conditions should be highlighted as well. It is apparent that human large synovial joints (hips, knees) which usually suffer from osteoarthritis, operate under transient load and kinematics. However, it should be mentioned that laboratory pin-on-plate reciprocating testing became a routine established technique when examining friction, lubrication, and wear mechanisms in biotribology area. However, variable change of load throughout the stroke, different length of individual strokes, or more complex eight-like or spiral-like motion might be a motivation for future research. This idea is supported by findings provided by Myant et al. [44], who described a clear effect of transient motion on lubrication behaviour of artificial joints.

## 5. Conclusion

The present study dealt with complex assessment of frictional behaviour of articular cartilage. The experiments were realized using pin-on-plate reciprocating tribometer while the contact of porcine femoral cartilage and smooth glass plate was studied. Thirteen model lubricants were compared, focusing on the role of concentration and mutual interaction of individual synovial fluid constituents. The experiments were carried out under two speeds corresponding to slow and normal walking. Furthermore, two levels of load mimicking walking and stair climbing were considered. The main findings are summarized in the following points.

- Focusing on the differences in concentration of fluid constituents corresponding to physiologic and osteoarthritic synovial fluid, only a limited effect on friction was observed when protein-based lubricants were applied.
- The lowest friction was generally observed for the mixture of γ-globulin, HA, and PHs, indicating the apparent role of interaction of the constituents. In addition, the importance of HA and PHs despite its lower concentration was demonstrated.
- The effect of HA and PHs can be further discussed in relation to sliding speed. The results showed that these constituents lead to stabilized friction with a negligible speed effect.
- An increased load led to lower friction, in general. This finding is in agreement with previous scientific observations.
- Regarding the implication for biotribological testing of articular cartilage, the importance of the interaction of proteins with HA and PHs should be highlighted. Therefore, it is strongly recommended to employ complex model fluids when examining cartilage behaviour.
- Further research should explore the role of transient kinematic and loading conditions, among other things [44]. In addition, the role of other constituents (e.g., lubricin) should be clarified, as it has been indicated in the literature that it may influence cartilage lubrication properties [25,26,45].

## Figures and Tables

**Table 1:** The combinations of the constituents in the lubricants.

Model Fluid Constituents					
1	PBS	—	—	—	—
2	PBS	Albumin	—	—	—
3	PBS	—	γ-globulin	—	—
4	PBS	Albumin	γ-globulin	—	—
5	PBS	Albumin	—	Hyaluronic acid	—
6	PBS	—	γ-globulin	Hyaluronic acid	—
7	PBS	Albumin	γ-globulin	Hyaluronic acid	—
8	PBS	Albumin	—	—	Phospholipids
9	PBS	—	γ-globulin	—	Phospholipids
10	PBS	Albumin	γ-globulin	—	Phospholipids
11	PBS	Albumin	—	Hyaluronic acid	Phospholipids
12	PBS	—	γ-globulin	Hyaluronic acid	Phospholipids
13	PBS	Albumin	γ-globulin	Hyaluronic acid	Phospholipids

**Table 2:** Used concentrations of the constituents [36].

Constituents	Physiologic Concentration	Osteoarthritic Concentration
	(mg/mL)	(mg/mL)
Albumin	20.00	24.90
γ-globulin	3.60	6.10
Hyaluronic Acid	2.50	1.49
Phospholipids	0.15	0.34
